# Emergency Department Buprenorphine Quality Improvement and Emergency Physician Knowledge, Attitudes, and Self-Efficacy

**DOI:** 10.5811/westjem.59477

**Published:** 2023-09-14

**Authors:** Michelle L. Myles, Kelli Scott, Hannah N. Ziobrowski, Sarah A. Helseth, Sara Becker, Elizabeth A. Samuels

**Affiliations:** * Brown University, Alpert Medical School, Department of Emergency Medicine, Providence, Rhode Island; † Northwestern University, Feinberg School of Medicine, Center for Dissemination and Implementation Science, Chicago, Illinois; ‡ Brown University, School of Public Health, Department of Epidemiology, Providence, Rhode Island; § University of California Los Angeles, Department of Emergency Medicine, Los Angeles, California

## Abstract

**Objective:**

Buprenorphine is an evidence-based treatment for opioid use disorder that is underused in the emergency department (ED). In this study we evaluated changes in emergency physician knowledge, confidence, and self-efficacy regarding buprenorphine prescribing and working with patients who use drugs after implementation of an ED buprenorphine quality improvement (QI) initiative.

**Methods:**

An anonymous, online survey was administered to emergency physicians staffing four EDs in New England in 2019 and 2020 before and after an ED QI initiative. Survey questions included novel and previously validated questions to assess confidence, knowledge, self-efficacy, and attitudes about buprenorphine and working with patients who use drugs. Confidence, self-efficacy, and attitude responses were assessed on a Likert scale. Participants received a gift card for survey completion. We analyzed pre- and post- survey responses descriptively and compared them using *t*-tests. Using logistic regression we evaluated the factors associated with buprenorphine prescribing.

**Results:**

Of 95 emergency physicians, 56 (58.9% response rate) completed the pre-intervention survey and 60 (63.2%) completed the post-survey. There was an increase in the number of X-waivered adult emergency physicians and ED buprenorphine prescribing after program implementation. Physician confidence increased from a mean of 3.4 (*SD* 0.8) to 3.9 (*SD* 0.7; scale 1–5, *p* < 0.01). Knowledge about buprenorphine increased from a mean score of 1.4 (*SD* 0.7) to 1.7 (*SD* 0.5, *p* < 0.01). Physician attitudes and self-efficacy did not change. Post-initiative, increased confidence was associated with higher odds of buprenorphine prescribing (odds ratio 4.4; 95% confidence interval 1.07–18.4).

**Conclusion:**

After an ED QI initiative, buprenorphine prescribing in the ED increased, as did both physician confidence in working with patients who use drugs and their knowledge of buprenorphine. Increased confidence was associated with higher odds of buprenorphine prescribing and should be a focus of future, buprenorphine implementation strategies in the ED.

## INTRODUCTION

More than one in 20 people treated in an emergency department (ED) after a non-fatal overdose will die within a year, and of those just over two-thirds die from an opioid-related overdose.^1^ Visits to the ED by patients with opioid use disorder (OUD) are an important opportunity to prevent overdose deaths and connect patients to evidence-based harm reduction services and treatment.

Buprenorphine treatment for OUD reduces mortality by over 50%^2^ ^,^ ^3^; however, a minority of people with OUD receive medication. Emergency department-initiated buprenorphine improves engagement in outpatient addiction treatment,^4^ is cost effective,^5^ and safe.^6^ ^,^ ^7^ Uptake, however, has lagged. Noted barriers include physician comfort in counseling patients and ordering buprenorphine, regulatory concerns, the need for additional training and supports, and robust referral upon ED discharge.^8^ ^,^ ^9^

Rhode Island has one of the highest rates of opioid overdose deaths in the United States.^10^ Since 2014, the Lifespan Opioid Overdose Prevention Program (LOOP) has worked to improve ED OUD care at Lifespan- affiliated EDs in Rhode Island.^11^ To increase ED-initiated treatment of OUD, in June 2019 LOOP launched an ED quality improvement (QI) initiative to provide buprenorphine for treatment of OUD and opioid withdrawal. We surveyed attending emergency physicians (EP) before and after implementation to assess changes in their knowledge, attitudes, behaviors, confidence, and self-efficacy regarding ED buprenorphine use. Basing our study on the theory of planned behavior (TPB),^12^ we hypothesized that after the initiative, EPs would report more knowledge, self-efficacy, and confidence, and more positive attitudes toward working with patients with OUD and prescribing buprenorphine in the ED.

## METHODS

### Procedures

This was an anonymous survey of EPs working in a Rhode Island hospital who were recruited through a faculty email listserv. Surveys were completed anonymously on a web platform (Qualtrics Provo, UT). The same survey was administered in June 2019 pre-intervention and again in May 2020 after implementation. Participants received a $20 gift card for each survey. This study was reviewed and deemed exempt by the Lifespan Institutional Review Board.

### Intervention

In June 2019, LOOP launched a buprenorphine QI initiative that included educational lectures at faculty retreat and residency conferences, a standardized buprenorphine-prescribing protocol posted in all clinician work areas, and on-shift prescribing support through a physician-staffed 24/7 support warm line. The ED buprenorphine protocol was finalized in October 2019. Clinician education was provided at residency conferences and faculty meetings and via email communications and signage within the ED. Clinician-facing signage included the ED buprenorphine treatment algorithm (Appendix 1). Support was offered through a 24/7 warm line staffed by EPs with ED buprenorphine and addiction medicine expertise.

At the time this study was conducted, clinicians interested in prescribing buprenorphine were required to obtain a federal X waiver to prescribe buprenorphine, which required them to attend an eight-hour Drug Addiction Treatment Act of 2000 (DATA 2000) training and submit an application to the Substance Abuse and Mental Health Services Administration. Attending a training was not required by the study ED, but those who attended waiver training and obtained an X waiver received a $150 incentive. (The waiver requirement has since been removed.^8^)

### Measures

We developed the survey questions in alignment with the domains of the TPB framework (Appendix 2). The questions included a combination of previously validated and study-specific items about caring for patients who use drugs^13^ and buprenorphine prescribing (Appendix 3). Validated questions about attitudes, confidence, willingness, and self-efficacy^13^ ^,^ ^14^ used a five-point Likert scale (1 = strongly disagree, 5 = strongly agree). Knowledge questions were multiple choice with multiple correct responses; each item was scored by summing total correct responses and subtracting total incorrect responses. We calculated total knowledge scores by summing individual knowledge scores. Attitudes, willingness, self-efficacy, knowledge, and confidence were scored by averaging responses across all domain items.

Participants were asked to rate patient, ED, and pharmacy characteristics as barriers or facilitators to ED buprenorphine prescribing on a 10-point Likert scale (1 = not a barrier or facilitator to 10 = significant barrier or facilitator). The survey was pilot tested with clinicians in the ED prior to distribution. We queried the electronic health record (EHR) to examine counts of ED-administered buprenorphine and discharge EPs working in the study EDs.

### Outcomes

Primary outcomes included changes in the domains of knowledge, self-efficacy, confidence, and attitudes post intervention. We also examined domains associated with reported buprenorphine prescribing.

### Data Analysis

We downloaded survey data from the web platform, excluding from our analysis any missing items. Survey responses and buprenorphine prescribing were analyzed descriptively. We ran independent samples *t*-tests in SPSS statistical software (SPSS Inc, Chicago, IL) to compare ED-wide changes across all domains. A Bonferroni correction was performed to account for multiple comparisons. We conducted independent sample *t*-tests in SPSS to compare pre-post mean differences in 13 barriers to prescription of buprenorphine in the ED. Bonferroni correction was performed to account for multiple comparisons.

Finally, we employed logistic regression to evaluate predictors of reported buprenorphine prescribing. Four models were run using pre-post initiative X-waiver attainment and pre-post buprenorphine prescription at patient discharge as dependent variables. Theorized predictors tested in the pre-initiative models included pre-implementation self-efficacy, confidence, attitudes, and knowledge. Predictors were identical for the post-implementation models. All logistic regression models controlled for completion of X-waiver training.

## RESULTS

### Study Subjects

Fifty-six of 95 attending physicians (58.9%) completed the pre-survey while 60/95 (63.2%) completed the post-survey. There were no missing responses in the pre-survey. Two respondents provided partial responses in the post-survey, one not completing knowledge and confidence questions and two not completing questions about attitudes and buprenorphine prescribing. Analysis was completed with all available data. Respondent pre- and post-survey age and gender demographics were similar (Table 1).

**Table 1.** Respondent demographics and characteristics.

	Pre-intervention n = 56 N (%)	Post-intervention n = 60 N (%)
Gender		
Male	33 (58.9)	28 (46.7)
Female	20 (35.7)	28 (46.7)
Transgender	0 (0)	0 (0)
Not reported	3 (5.3)	11 (18.3)
Age		
20–30 years	2 (3.6)	0 (0)
31–40 years	18 (32.1)	19 (31.7)
41–50 years	19 (33.9)	20 (33.3)
50+ years	15 (26.8)	17 (28.3)
Not reported	2 (3.6)	11 (18.3)
DATA 2000 X-waiver training		
Yes	27 (48.2)	49 (81.7)
No	29 (51.8)	10 (16.7)
Not reported	0 (0)	8 (13.3)
X-waivered	17 (30.4)	42 (70.0)
Ever prescribed buprenorphine upon discharge from ED		
Yes	*4 (7.1)*	*23 (38.3)*
No	*44 (78.6)*	*33 (55.0)*
No, but had someone else	*8 (14.3)*	*2 (3.3)*
Prescribe Missing	*0 (0)*	*9 (15.0)*

*DATA 2000*, Drug Addiction Treatment Act of 2000.

### Pre-Survey Results

Almost half (27/56) of respondents had completed the DATA 2000 waiver training pre-intervention. Of those, only 62.9% (17/27) had received their X-waiver. Respondents reported moderate self-efficacy (mean 3.5 [SD 0.5], scale 1–5) and confidence (mean 3.4 [SD 0.8], scale of 1–5) in caring for people who use drugs. Attitudes were positive, with an average score of 3.8 ([SD 0.8], scale 1–5). Over half (31/56) reported ever administering buprenorphine in the ED; however, only 23.5% (4/17) of X-waivered physicians reported ever prescribing buprenorphine upon discharge. The most selected barriers included patient disinterest in treatment (mean 6.7 [SD 2.7], scale 1-10), availability of outpatient services (mean 5.9 [SD 3.1], scale 1–10), comfort with counseling patients (mean 5.8 [SD 2.9], scale 1–10), lack of knowledge (mean 5.8 [SD 3.1], scale 1–10), and time constraints (mean 5.7 [SD 3.0], scale 1–10). The most selected facilitators included pre-packaged prescription kits (mean 7.0 [SD 3.5], scale 1–10) and presence of an ED-based OUD patient engagement program (mean 6.4 [SD 3.7], scale 1–10). Per EHR data, in 2019 there were 48 prescriptions written for buprenorphine and monthly prescriptions ranged from two to eight (median 4).

### Post-Survey Results

Sixty of 95 (63.2%) attendings completed the post-survey. The proportion of respondents who completed X-waiver training increased to 81.7% (60), and the proportion receiving their X-waiver increased from 30.4% (17/56) to 70.0% (42/60) after our intervention. Reported buprenorphine prescribing also increased from 7.1% in 2019 to 38.3% in 2020. Physician confidence increased from a mean of 3.4 (SD 0.8) to 3.9 (SD 0.7; scale 1–5, *P* < 0.01). Overall knowledge was unchanged; however, knowledge about ED buprenorphine use increased from a mean score of 1.4 (SD 0.7) to 1.7 (SD 0.5, *P* < 0.01) (Table 2). Physician attitudes and self-efficacy did not change (Table 2). There were 98 buprenorphine prescriptions in 2020 (median seven monthly prescriptions, range 5–22), almost doubling from 2019. Independent sample *t*-tests were run to evaluate differences in 13 barriers to buprenorphine prescription prior to and post initiative. After performing a Bonferroni correction we found no significant pre-post differences in barriers to buprenorphine prescription.

**Table 2.** Self-efficacy, confidence, attitudes, and knowledge before and after ED intervention (scale 1 to 5) (pre: N = 56; post: N = 60).

	Pre-intervention	Post-intervention
	Mean (SD)	Mean (SD)
Self-efficacy	3.5 (0.5)	3.5 (0.7)
Confidence	3.4 (0.8)	3.9 (0.7)
Attitudes	3.8 (0.8)	3.8 (0.8)
Overall knowledge		
Mean (SD)	11.6 (2.0)^1^	12.3 (2.1)^1^
Range	8 to 15	5 to 15
ED buprenorphine knowledge		
Mean (SD)	1.4 (0.7)^2^	1.7 (0.5)^2^
Range	−1 to 2	1 to 2

^1^ Differences were not statistically significant.

^2^ *P* < 0.01.

### Logistic Regression Results

Physician confidence was a predictor for both pre- and post -implementation buprenorphine prescribing. Before the intervention, clinician confidence was associated with lower odds of buprenorphine prescribing (odds ration [OR] 0.3; 95% confidence interval [CI] 0.1, 0.9). After the intervention, confidence was a significant predictor of buprenorphine prescribing, such that the odds of prescribing increased more than four times (OR 4.4; 95% CI 1.1, 18.4) for a one-unit increase in physician confidence.

## DISCUSSION

Guided by the TPB,^10^ we sought to capture changes in EPs’ knowledge of, attitudes toward, and confidence in prescribing buprenorphine following an ED buprenorphine QI initiative. Physicians demonstrated improvements in their knowledge about and confidence in prescribing buprenorphine and treating patients with OUD, while changes in attitudes and self-efficacy were not observed. Previous studies have noted a similar discrepancy between knowledge about buprenorphine and prescribing comfort.^15^ Improvements in confidence were observed alongside increases in monthly buprenorphine prescriptions. We also significantly expanded the number of waivered physicians with fewer monetary incentives compared to other institutions, which might suggest that other institutional supports such as education and protocols may be more likely to encourage physicians to provide buprenorphine in the ED.^16^

Identified barriers to ED-administered buprenorphine included need for physician consultation, lay ED overdose engagement specialists, time constraints, comfort in counseling patients, and knowledge. Previous studies have identified similar barriers to prescribing, including the former X-waiver requirement.^8^ ^,^ ^17^ By addressing these and other environmental factors, EPs had the resources and institutional support they needed to successfully change their prescribing behavior. Such organization-wide initiatives are vital to increasing access to evidence-based treatments for OUD.

## LIMITATIONS

This study has several limitations. Participant identifiers were not collected; thus, we were unable to link pre- and post-implementation responses, limiting our ability to assess individual changes in attitudes and behaviors over time. Our sample was small and consisted of academic EPs working in Rhode Island; thus, our findings may not be generalizable to other regions or hospital types. Finally, the survey did not capture attitudes, knowledge, and confidence of advanced practice practitioners or resident physicians.

## CONCLUSION

Removal of the federal X-waiver requirement will lower barriers to ED buprenorphine prescribing; however, educational and institutional initiatives are needed to reduce prescribing barriers and improve physician confidence in prescribing. Given the increased odds of buprenorphine prescribing with higher confidence, improving physician confidence may be an important target for future buprenorphine-implementation strategies in the ED. Future efforts are needed to improve physician skills, self-efficacy, and attitudes and to continue to minimize barriers to buprenorphine prescribing.

## Supplementary Information






