# Supplementary material for: Emergency Department Buprenorphine Quality Improvement and Emergency Physician Knowledge, Attitudes, and Self-Efficacy
Source: West J Emerg Med. 2023 Sep 14;24(6):1005–9. doi: 10.5811/westjem.59477 (PMC10754198; doi:10.5811/westjem.59477)
Supplement: Supplementary file 1 [file wjem-24-1005-s001.pdf]

Lifespan Opioid Overdose Prevention Program

Buprenorphine Algorithm

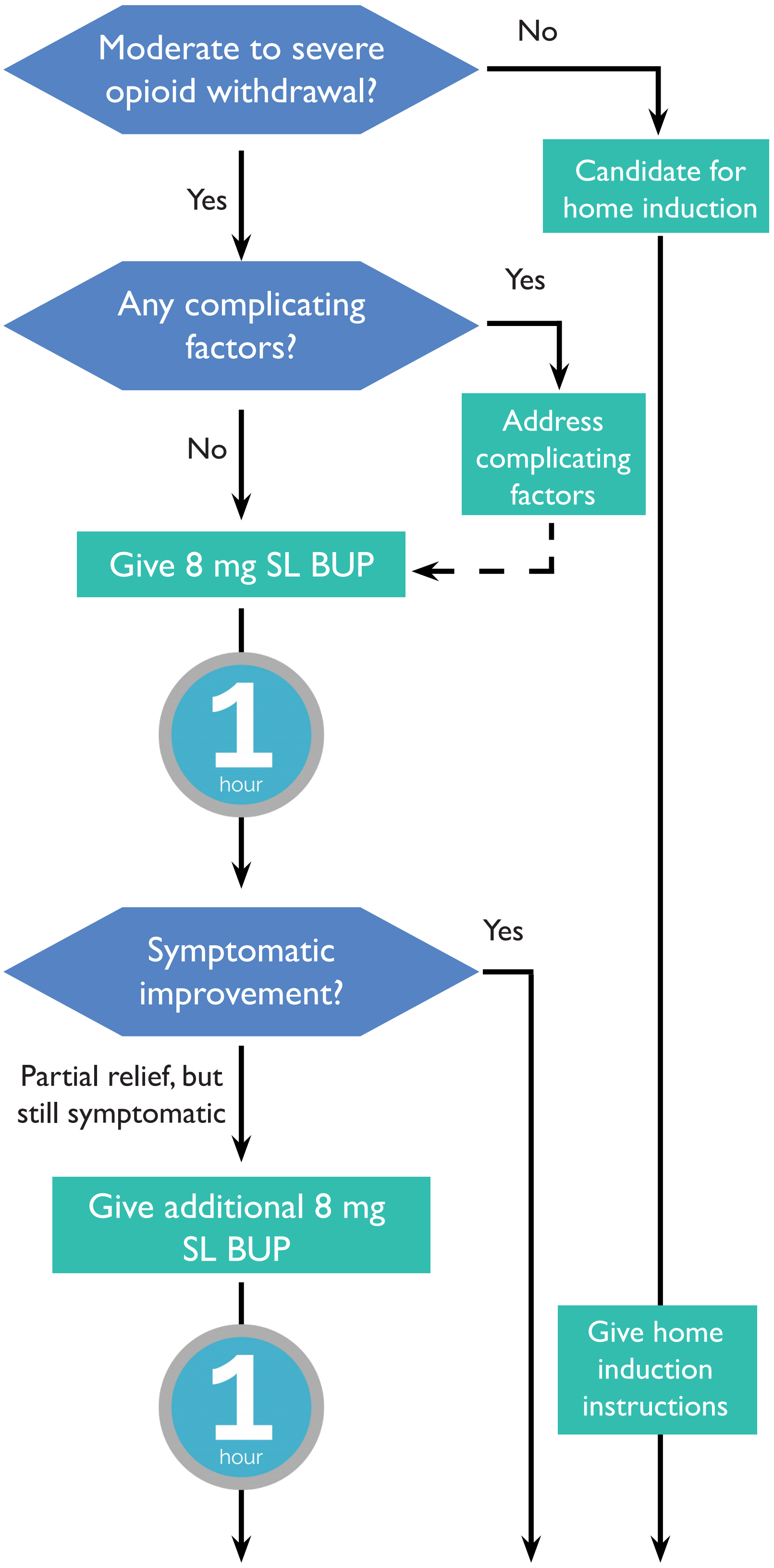

Discharge with:

1. Rx: buprenorphine-naloxone (Suboxone) 8mg **twice** a day for **7** days (if waivered)
2. Take-home IN Naloxone
3. Ambulatory referral to Lifespan Recovery Center

Questions?

Call the BEM ED buprenorphine on-call provider:

(401) 830-0767

For use in adult ED patients with moderate to severe opioid use disorder (OUD) being discharged

- Moderate to severe OUD = daily opioid use and onset of withdrawal when opioids are not used

MODERATE TO SEVERE OPIOID WITHDRAWAL

- Use clinical judgment or the Clinical Opioid Withdrawal Scale (COWS)
- Can give buprenorphine if COWS ≥ 8 with 1 objective sign of withdrawal
- Document: Which opioid used, last time used

COMPLICATING FACTORS

DO NOT give buprenorphine if patient:

- Has altered mental status or is intoxicated
- Is medically unstable
- Is on methadone

Talk to on-call Brown EM Buprenorphine provider if:

- Has OUD but withdrawal inconsistent or borderline

Pregnant or breastfeeding patients should be referred to the WIH Moms MATTER clinic: 401-430-2700. Provide patient name & contact info, clinic case manager will call the patient within 24h

BUPRENORPHINE DOSING

- Redose buprenorphine if patients improved after first dose but still having withdrawal symptoms
  - Target dose 8-16mg, sufficient for most
  - If needs more, max dose in ED 32mg

PRECIPITATED WITHDRAWAL

- Decreased risk with longer time since last opioid use and greater withdrawal symptoms
- Treat with additional buprenorphine

REFERRAL TO TREATMENT:

- If waivered, prescribe buprenorphine-naloxone 16mg a day for 3 days
- Refer patients for an appointment in 24-48h
  - Ambulatory Referral to Lifespan Recovery Center
  - See treatment center list
- *If not waivered*, and no next day appointment, patient can come back to ED next day for additional dose

EPIC:

- Use Naloxone Order Set
  - Includes orders for IN Naloxone and ambulatory referral to Lifespan Recovery Center
- Check PDMP (*required for all controlled substances*)

In chart, use (if applicable):

- Overdose dotphrase: .uemfopioid
- Buprenorphine dotphrase: .bembupnote

Patient Instructions

- Buprenorphine patient instructions: .bembupdc
- Home induction discharge instructions (*if applicable*)

RESOURCES:

- ACEP BUPE: https://www.acep.org/patient-care/bupe/
- UCSF Substance Use Warmline: 855-300-3595, 10 am – 6 pm EST Monday - Friday

Adapted with permission from: ED BRIDGE, CALIFORNIA ACEP

updated 7.30.2019
