# Supplementary figures and images for: Emergency Department Buprenorphine Quality Improvement and Emergency Physician Knowledge, Attitudes, and Self-Efficacy
Source: West J Emerg Med. 2023 Sep 14;24(6):1005–9. doi: 10.5811/westjem.59477 (PMC10754198; doi:10.5811/westjem.59477)

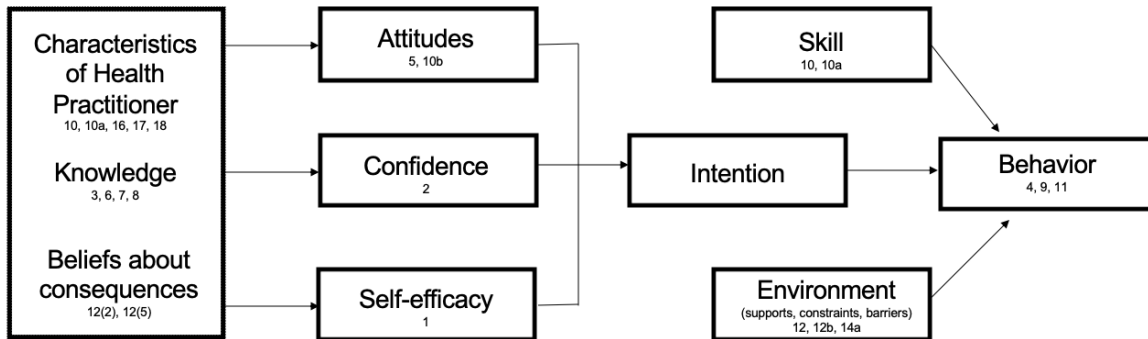

Supplement: Supplementary file 2 [file wjem-24-1005-s002.pdf]
