# Supplementary material for: Emergency Department Buprenorphine Quality Improvement and Emergency Physician Knowledge, Attitudes, and Self-Efficacy
Source: West J Emerg Med. 2023 Sep 14;24(6):1005–9. doi: 10.5811/westjem.59477 (PMC10754198; doi:10.5811/westjem.59477)
Supplement: Supplementary file 3 [file wjem-24-1005-s003.docx]

**Physician Buprenorphine Survey**

Elizabeth A. Samuels, MD, MPH, MHS

Based on a theory of planned behavior framework, I developed a survey instrument to assess emergency medicine prescriber knowledge, attitudes, behaviors, and barriers to providing buprenorphine in the emergency department. The theoretical model (Figure 1) and survey instrument (below references) are adapted from existing theory of planned behavior theoretical models^1-3^ and prior physician and addition medicine surveys.^3-5^

Survey questions align with each domain of the above theoretical model. The goal of the survey is to identify potential areas for behavioral intervention and measure changes in each domain after implementing an ED buprenorphine protocol using multimodal education and provider engagement strategies. The pre-survey was distributed in June 2019. The post-survey was distributed in May 2020. During the implementation year, we provided faculty education through educational conference sessions, presentations at the required annual faculty retreat, emails, and posted protocols in the provider work spaces. Provider support was made available through a physician ‘warmline’ to answer questions about providing buprenorphine. Smartphrases were developed and disseminated to aid in electronic health record documentation and patient education when buprenorphine was prescribed in the ED. Finally, providers were offered financial incentives for completion of the DATA 2000 waiver course.

Survey analysis will entail comparison each domain between the pre- and post- implementation surveys. Regression analysis will also be conducted to identify factors that are associated or not-associated with reported buprenorphine prescribing, the desired outcome. This will help identify areas for intervention including, but not limited to, provider education, provider self-efficacy, barrier removal, and establishment of interventions to provide more provider support. The summary table below provides an outline of how survey responses fit within each domain.

| Survey Response Summary Table | |  |
| --- | --- | --- |
| Self-Efficacy working with patients with addiction *(scale 1-5)* | Mean [95% CI] | |
| Composite Score |  | |
| Little can do to help |  | |
| Able to work with |  | |
| I am a failure |  | |
| Disrespect |  | |
| Uncomfortable |  | |
| Can get satisfaction |  | |
| Rewarding to work with |  | |
| I understand |  | |
| Confidence *(scale 1-5)* | Mean [95% CI] | |
| Composite Score |  | |
| Engage patients following an opioid overdose |  | |
| Provide brief substance use counseling |  | |
| Identify patients at risk for opioid overdose |  | |
| Counsel patients about medication for opioid use disorder (OUD) |  | |
| Administer/prescribe buprenorphine in the ED |  | |
| Link patients to outpatient addiction treatment |  | |
| Attitudes *(scale 1-5)* | N (% [95% CI]) | |
| *Recovery coaches* |  | |
| Help facilitate linkage to treatment |  | |
| Come in a timely manner |  | |
| Are a barrier to evidence-based addiction treatment |  | |
| *Waiver Training* |  | |
| Improved understanding of OUD |  | |
| Ability to engage patients with OUD |  | |
| Ability to engage patients after an opioid overdose |  | |
| Knowledge about treatment of OUD |  | |
| Comfort starting patients on buprenorphine |  | |
| Behavior | N (% [95% CI]) | |
| Ever prescribed buprenorphine in the ED |  | |
| Ever written a discharge prescription for buprenorphine |  | |
| *Routinely provide for opioid overdose patients:* |  | |
| Dispense take-home naloxone rescue kit in the ED |  | |
| Patient assessment for readiness to seek treatment |  | |
| Discharge prescription for naloxone |  | |
| Anchor Recovery Coach Consult |  | |
| Referral to an outpatient treatment program/provider |  | |
| Patient education about overdose prevention |  | |
| HIV testing |  | |
| Referral to syringe access program |  | |
| Knowledge *(scale 1-5)* | Mean [95% CI] | |
| Evidence based treatment for OUD |  | |
| Use of buprenorphine for opioid use disorder |  | |
| ED buprenorphine evidence |  | |
| Patient eligibility for ED buprenorphine |  | |
| Barriers to Buprenorphine Prescribing *(scale 1-5)* | Mean [95% CI] | |
| Time constraints |  | |
| Lack of knowledge about prescribing buprenorphine |  | |
| Comfort with counseling patients about buprenorphine |  | |
| Availability of outpatient substance use treatment services after discharge |  | |
| Consultation with Social Work |  | |
| Consultation with a Recovery Coach |  | |
| Resistance encountered from nursing staff |  | |
| Resistance encountered from pharmacy |  | |
| Lack of patient interest in treatment |  | |
| Patient preference for other types of treatment |  | |
| Supports for Buprenorphine Prescribing *(scale 1-5)* | Mean [95% CI] | |
| Clinical decision support in Epic |  | |
| Availability of pharmacist consultation |  | |
| Availability of physician consultation |  | |
| Pre-packaged prescriptions 'kits' with 4-days supply of buprenorphine |  | |
| Lay ED overdose/OUD engagement specialist to coordinate care |  | |
| Beliefs about Consequences of Buprenorphine *(scale 1-5)* | Mean [95% CI] | |
| Deaths due to opioid overdose |  | |
| Diversion |  | |

**References**

1. Limbert C, Lamb R. Doctors' use of clinical guidelines: Two applications of the Theory of Planned Behaviour. *Psychology, Health & Medicine.* 2010;7(3):301-310.

2. Godin G, Belanger-Gravel A, Eccles M, Grimshaw J. Healthcare professionals' intentions and behaviours: a systematic review of studies based on social cognitive theories. *Implement Sci.* 2008;3:36.

3. Samuels EA, Dwyer K, Mello MJ, Baird J, Kellogg A, Bernstein E. Emergency Department-Based Opioid Harm Reduction: moving physicians from willing to doing. *Acad Emerg Med.* 2016;23(4):455-465.

4. Watson H, Maclaren W, Kerr S. Staff attitudes towards working with drug users: development of the Drug Problems Perceptions Questionnaire. *Addiction.* 2007;102(2):206-215.

5. Lowenstein M, Kilaru A, Perrone J, et al. Barriers and facilitators for emergency department initiation of buprenorphine: A physician survey. *Am J Emerg Med.* 2019;37(9):1787-1790.

ED Buprenorphine Prescriber Survey

Start of Block: Default Question Block

This CONFIDENTIAL & ANONYMOUS survey examines Brown Emergency Medicine providers’ knowledge, attitudes, and barriers to treatment of opioid use disorder (OUD) and medication for OUD.

**This survey should take 10-15 minutes of your time.** It is part of a research study and your participation in this study is completely voluntary. You have the right to not complete the survey and you have the right not to answer questions you prefer not to answer. If you choose not to participate, it will not impact your job in any way.

No individually identifiable information will be linked to your survey responses or published in any written study summaries or reports. All data will be analyzed and presented in aggregate. There are no significant risks to participation and while you will receive no personal benefit from taking this survey, your participation may help others.

This survey is being conducted as part of a Lifespan IRB approved research study. Information will be used for program evaluation and improvement and survey results will be reported in aggregate in peer-reviewed journals.   If you have questions about the study, you can contact the study principle investigator Dr. Elizabeth Samuels at (401) 444-8867, elizabeth_samuels@brown.edu. If you have any questions about your rights as a research subject, please contact Janice Muratori in the Office of Research Administration at (401) 444-6246 or jmuratori@lifespan.org.

By completing and submitting this survey you acknowledge that you have been informed of your rights as a human subject, and that you consent to participate in this research study.

**You will receive a $25 Amazon gift card for completing this survey.**

If you have read the above informed consent and agree to take this survey, select the consent option below and click the arrow to begin.

- I understand the risks and benefits associated with this survey and give full consent to participate. (1)
- I decline to participate. (2)

Skip To: 1 If = I understand the risks and benefits associated with this survey and give full consent to participate.

Skip To: 1 If = I understand the risks and benefits associated with this survey and give full consent to participate.

| 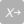 | 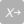 |
| --- | --- |

1 There are a range of perspectives about working with patients with addictions. For each statement below, select your level of agreement:
*(select one answer choice for each statement)*

|  | Strongly agree (1) | Agree (2) | Neither agree nor disagree (3) | Disagree (4) | Strongly disagree (5) |
| --- | --- | --- | --- | --- | --- |
| I feel that there is little I can do to help people who use drugs. (1_1) |  |  |  |  |  |
| I feel that I am able to work with people who use drugs as well as other patient groups. (1_2) |  |  |  |  |  |
| All in all I am inclined to feel that I am a failure with people who use drugs. (1_3) |  |  |  |  |  |
| In general, I have less respect for people who use drugs than for most other patients I work with. (1_4) |  |  |  |  |  |
| I often feel uncomfortable when working with people who use drugs. (1_5) |  |  |  |  |  |
| In general, one can get satisfaction from working with people who use drugs. (1_6) |  |  |  |  |  |
| In general, it is rewarding to work with people who use drugs. (1_7) |  |  |  |  |  |
| In general, I feel I can understand people who use drugs. (1_8) |  |  |  |  |  |

| Page Break |  |
| --- | --- |

2 How CONFIDENT are you in your ability to do the following: 
*(select one answer choice for each statement)*

|  | Not at all confident (1) | Lacking Confidence (2) | Unsure (3) | Somewhat confident (4) | Very Confident (5) |
| --- | --- | --- | --- | --- | --- |
| Engage patients following an opioid overdose (1) |  |  |  |  |  |
| Provide brief substance use counseling (2) |  |  |  |  |  |
| Identify patients at risk for opioid overdose (6) |  |  |  |  |  |
| Counsel patients about medication for opioid use disorder (OUD) (3) |  |  |  |  |  |
| Administer/prescribe buprenorphine in the ED (4) |  |  |  |  |  |
| Link patients to outpatient addiction treatment (5) |  |  |  |  |  |

3 Which of the following are effective, evidence-based treatments for opioid use disorder (alone or in combination)? *(select all that apply)*

- Detox (1)
- Methadone (2)
- Behavioral Counseling (3)
- Buprenorphine (4)
- Peer to peer recovery support (5)
- Abstinence-only treatment (6)
- Naltrexone (7)

| Page Break |  |
| --- | --- |

**The following questions about services currently provided in Lifespan Emergency Departments.**

| 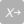 |
| --- |

4 Which of the following are part of your routine care for opioid overdose patients? *(select all that apply)*

- Social Work Consult (1)
- Dispense take-home naloxone rescue kit in the ED (2)
- Patient assessment for readiness to seek treatment (3)
- Discharge prescription for naloxone (4)
- Anchor Recovery Coach Consult (5)
- Referral to an outpatient treatment program/provider (6)
- Patient education about overdose prevention (7)
- HIV testing (8)
- Referral to syringe access program (9)

| Page Break |  |
| --- | --- |

| 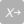 | 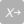 |
| --- | --- |

5 Please indicate how much you AGREE with the following statements about the Anchor Peer Recovery Coaches.
 *(select one answer choice for each statement)*

|  | Strongly Disagree (1) | Disagree (2) | Neither Agree Nor Disagree (3) | Agree (4) | Strongly Agree (5) |
| --- | --- | --- | --- | --- | --- |
| Recovery Coaches help facilitate linkage to outpatient treatment. (9_1) |  |  |  |  |  |
| Recovery Coaches come in a timely manner. (9_2) |  |  |  |  |  |
| Recovery Coaches are a barrier to evidence-based addiction treatment. (9_3) |  |  |  |  |  |

| Page Break |  |
| --- | --- |

**The following questions are about treatment of opioid use disorder (OUD) and/or opioid withdrawal with buprenorphine.**
 
*Buprenorphine is typically prescribed in combination with naloxone: buprenorphine-naloxone (Suboxone). This survey will refer to this medication simply as buprenorphine.*

| 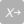 |
| --- |

6 Which of the following are true about opioid use disorder (OUD) treatment with buprenorphine: *(select all that apply)*

- Reduces incidence of opioid overdose (1)
- Reduces use of illicit opioids (2)
- Associated with increased mortality (3)
- Associated with increased treatment retention (4)
- It can precipitate opioid withdrawal (5)
- Anyone can prescribe buprenorphine (6)

7 ED initiation of buprenorphine in appropriate patients has been shown to: *(select all that apply)*

- Improve outpatient addiction treatment follow up in 30 days (1)
- Increase frequency of ED precipitated withdrawal (2)
- Be cost-effective (3)
- Reduce mortality (4)
- Reduce opioid overdose (5)

8 Patients given a dose of buprenorphine in the ED should meet which of the following requirements: *(select all that apply)*

- Have physiologic dependency on opioids (1)
- Are not on long acting opioids (i.e. methadone) (2)
- Are medically stable (3)
- Are in opioid withdrawal (Clinical Opioid Withdrawal Scale ≥8) (4)
- Have not previously been in addiction treatment (5)
- Have health insurance (6)

| Page Break |  |
| --- | --- |

| 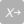 |
| --- |

9 Have you ever given buprenorphine in the ED? *(select one)*

- Yes (1)
- No (0)

| 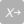 |
| --- |

10 Have you completed the DATA 2000 Waiver (aka X-waiver or buprenorphine waiver) training?  *(select one)*

- Yes (1)
- No (0)

Display This Question:

If 14 = Yes

| 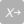 |
| --- |

Q10a After the training, did you receive your X-waiver? *(select one)*

- Yes (1)
- No (0)
- Don't Know (2)

Display This Question:

If 14 = Yes

| 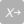 | 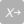 |
| --- | --- |

Q10b To what degree did the waiver training improve:
*(select one answer choice for each statement)*

|  | None at all (1) | A little (2) | | A moderate amount (3) | | A lot (4) | | A great deal (5) | |  |
| --- | --- | --- | --- | --- | --- | --- | --- | --- | --- | --- |
| Your understanding of opioid use disorder (OUD) (14b_1) |  | |  | |  | |  | |  | |
| Your ability to engage patients with OUD (14b_2) |  | |  | |  | |  | |  | |
| Your ability to engage patients after an opioid overdose (14b_3) |  | |  | |  | |  | |  | |
| Your knowledge about treatment of OUD (14b_4) |  | |  | |  | |  | |  | |
| Comfort starting patients on buprenorphine (14b_5) |  | |  | |  | |  | |  | |

| 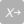 |
| --- |

11 Have you ever prescribed buprenorphine upon discharge from the ED?

- Yes (1)
- No (0)
- No, but I had another provider prescribe buprenorphine for my patient (2)

| Page Break |  |
| --- | --- |

**Barriers to buprenorphine**

12 To what degree are the following barriers to ordering buprenorphine for opioid withdrawal and/or treatment of opioid use disorder in the ED:(select one answer choice for each statement)

|  | Not a Barrier (1) | (2) | Neutral (3) | (4) | Significant Barrier (5) |
| --- | --- | --- | --- | --- | --- |
| Time constraints (1) |  |  |  |  |  |
| Concerns about medication safety or adverse effects (2) |  |  |  |  |  |
| Lack of knowledge about prescribing buprenorphine (3) |  |  |  |  |  |
| Comfort with counseling patients about buprenorphine (4) |  |  |  |  |  |
| Availability of outpatient substance use treatment services after discharge (5) |  |  |  |  |  |
| Regulatory concerns related to prescribing buprenorphine (6) |  |  |  |  |  |

| Page Break |  |
| --- | --- |

**Barriers to buprenorphine (continued)**

12b To what degree are the following barriers to ordering buprenorphine for opioid withdrawal and/or treatment of opioid use disorder in the ED: (select one answer choice for each statement)

|  | Not a Barrier (1) | (2) | Neutral (3) | (4) | Significant Barrier (5) |
| --- | --- | --- | --- | --- | --- |
| Consultation with Social Work (1) |  |  |  |  |  |
| Consultation with a Recovery Coach (2) |  |  |  |  |  |
| Resistance encountered from nursing staff (3) |  |  |  |  |  |
| Resistance encountered from pharmacy (4) |  |  |  |  |  |
| Concern about diversion or misuse of buprenorphine (5) |  |  |  |  |  |
| Lack of patient interest in treatment (6) |  |  |  |  |  |
| Patient preference for other types of treatment (7) |  |  |  |  |  |

13 Other barriers to prescribing buprenorphine not listed above:

________________________________________________________________

________________________________________________________________

________________________________________________________________

________________________________________________________________

________________________________________________________________

| Page Break |  |
| --- | --- |

14 Please indicate the how effective each of the following would be to increase your likelihood to administer and/or prescribe buprenorphine: (select one answer choice for each statement)

|  | Not effective at all (1) | (2) | Moderately effective (3) | (4) | Extremely effective (5) |
| --- | --- | --- | --- | --- | --- |
| Clinical decision support in Epic (1) |  |  |  |  |  |
| Availability of pharmacist consultation (2) |  |  |  |  |  |
| Availability of physician consultation (3) |  |  |  |  |  |
| Pre-packaged prescriptions 'kits' with 4-days supply of buprenorphine (5) |  |  |  |  |  |
| Lay ED overdose/OUD engagement specialist to coordinate care (6) |  |  |  |  |  |

15 What else do you think would assist with ED buprenorphine prescribing?

________________________________________________________________

________________________________________________________________

________________________________________________________________

________________________________________________________________

________________________________________________________________

| Page Break |  |
| --- | --- |

16 What is your Gender? *(select one)*

- Male (1)
- Female (2)
- Transgender (3)
- Not Listed (4) ________________________________________________

17 What is your age? *(select one)*

- 20-30 years of age (1)
- 31-40 years of age (2)
- 41-50 years of age (3)
- 51 years of age and older (4)

| 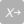 |
| --- |

18 Where do you work? *(check all that apply)*

- Hasbro Children's Hospital (1)
- Miriam Hospital (2)
- Newport Hospital (3)
- Rhode Island Hospital (4)

19 Please share any additional thoughts, comments, or suggestions:

________________________________________________________________

________________________________________________________________

________________________________________________________________

________________________________________________________________

________________________________________________________________

End of Block: Default Question Block
